# Infant and Young Child Feces Management and Enabling Products for Their Hygienic Collection, Transport, and Disposal in Cambodia

**DOI:** 10.4269/ajtmh.15-0423

**Published:** 2016-02-03

**Authors:** Molly K. Miller-Petrie, Lindsay Voigt, Lyn McLennan, Sandy Cairncross, Marion W. Jenkins

**Affiliations:** Faculty of Infectious and Tropical Diseases, London School of Hygiene and Tropical Medicine, London, United Kingdom; Water, Sanitation and Hygiene Enterprise Development, Phnom Penh, Cambodia; Civil and Environmental Engineering, University of California Davis, Davis, California

## Abstract

In Cambodia, children's feces are rarely disposed of in an improved sanitation facility. This study examines current practices and the role that enabling products may play in increasing hygienic management of infant and young child (IYC) feces in households with access to improved sanitation. A survey was conducted with the primary caregiver of a child under 5 years of age in 130 homes with an improved latrine in 21 villages across two provinces in Cambodia. Two focus group discussions per province were conducted after the survey to obtain caregiver feedback on new enabling products for hygienic management. Among caregivers, 63% reported child feces disposal in an improved latrine but only 36% reported doing so consistently. Besides child age, years of latrine ownership, caregiver age, consistency of adult latrine use, and presence of child feces management tools in the latrine were associated with hygienic disposal. The youngest caretakers with the newest latrines and youngest children were least likely to dispose of IYC feces hygienically, representing a key target group for interventions to improve hygienic disposal in Cambodia. Reusable diapers, child-friendly potties, and possibly latrine seats, that offer child safety, time and cost savings, and easy disposal and cleaning could potentially facilitate hygienic disposal for these ages.

## Introduction

Diarrheal disease is the second greatest cause of mortality for children under 5 years of age worldwide, accounting for nearly 800,000 deaths a year, primarily in developing countries.^1^

Among water, sanitation, and hygiene interventions for its prevention, the hygienic management of children's feces has received less attention as a target behavior to reduce diarrheal transmission, though it has been identified as one of the three “key water-related behaviors for promotion.”^2^ Children's feces are more likely to contain enteric pathogens than those of adults,^3^ and open defecation by young children contaminates the household environment, a key site for diarrheal disease transmission to children and adults.^4^

A systematic review of children's sanitation practices found that non-hygienic feces disposal increased the risk of diarrheal disease by 23% (risk ratio: 1.23, 95% confidence interval [CI]: 1.15–1.32) compared with hygienic behaviors, such as use of a latrine, potty, or diaper.^3^ In a subsequent update to the systematic review, results were “strongly suggestive of a protective effect of hygienic practice” (B. Scott, unpublished data).

In Cambodia, diarrheal incidence in children tends to be higher than that in the southeast Asian region as a whole. In 2010, those aged 6–11 and 12–23 months had the highest average annual incidences: 4.26 episodes (Cambodia) versus 3.7 episodes and 3.45 episodes versus three episodes, respectively, in the region.^5^

While 37% of all Cambodian households (and 25% of rural households) have access to an improved latrine,^6^ only 20% of the feces of children under 5 years of age were disposed of into an improved latrine.^7^ A recent evaluation in rural Cambodia showed a high level of reported use of newly purchased improved latrines by adults (96–97%), but a lower reported rate of use among children under 5 years of age (84–86%).^8^ Rates of safe child feces disposal in an improved sanitation facility are also very low in other south and southeast Asian countries.^9^–^11^

To improve the health of children under 5 years of age, barriers to safe child feces disposal must be addressed. One strategy is to encourage the use of well-designed, low-cost products to facilitate more hygienic management of infant and child feces. This study aimed to assess factors associated with hygienic disposal and caregiver interest in child sanitation management products.

## Materials and Methods

Six steps were identified in the management of infant and young child (IYC) feces for disease prevention as described in Table 1.

Data were collected in two stages. In stage 1, a household survey was conducted with the primary caregiver in 130 households comprising 145 children under 5 years of age from 21 villages in districts in Kampong Speu and Battambang provinces, Cambodia, where the nongovernmental organization Water, Sanitation and Hygiene Enterprise Development (WaterSHED) works to increase access to and use of improved sanitation. In stage 2, four focus group discussions (FGDs) were conducted with surveyed caretakers in two sampled villages in each province.

### Ethics approval.

Ethics approval for the study (London School of Hygiene and Tropical Medicine Control of Infectious Diseases [LSHTM CID] MSc project 107631) was obtained from the National Ethics Committee for Health Research, Ministry of Health, Cambodia, and from the LSHTM Ethics Review Committee before the start of fieldwork.

### Survey sampling and data collection.

Provinces and villages were purposefully selected for diversity in terms of water sources, socioeconomic status, geographical setting, and proximity to major roads and markets to sample across a wide range of potential barriers and facilitators to hygienic disposal. Kampong Speu is one of Cambodia's poorer provinces while Battambang is one of its wealthier provinces. To be considered, villages were required to have improved sanitation coverage of at least 80% and be located in WaterSHED program areas. Although WaterSHED was working in over 2,100 villages in these two provinces in 2014, only 205 met the sampling eligibility criteria for the study.

**Figure 1.**
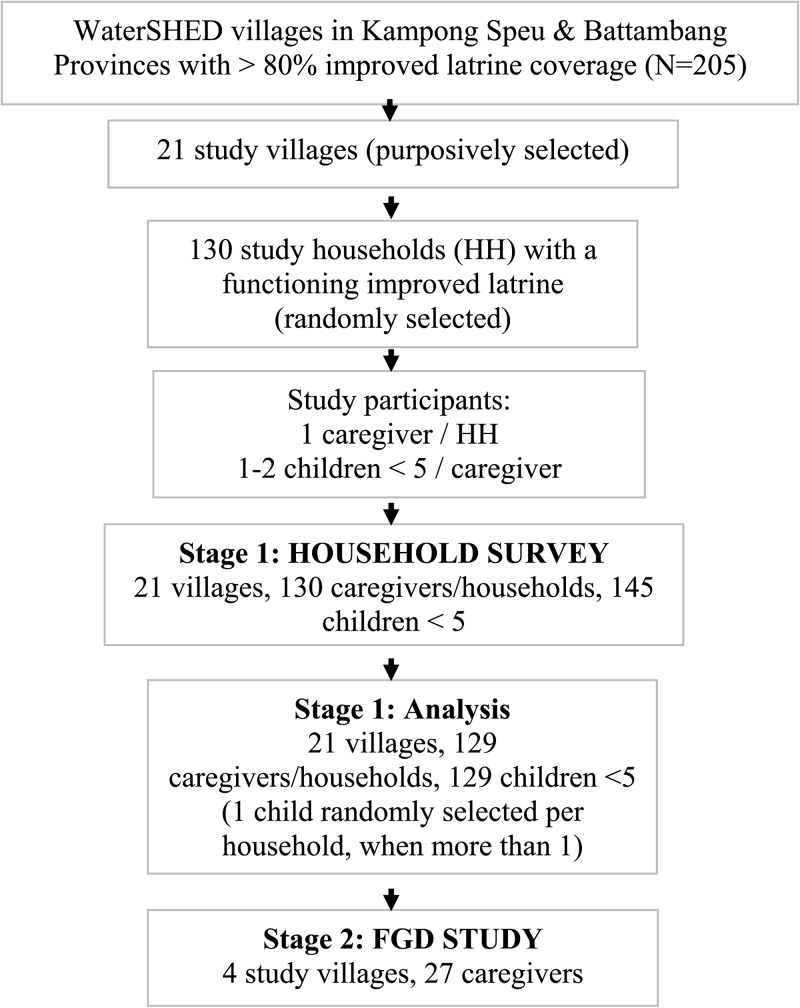
Sampling and study design overview.

Up to eight eligible households per village were selected using a random walk procedure for a total of 130 enrolled households. To be eligible, households had to own and use an improved latrine and have at least one child under 5 years of age. Participants had to be over 18 years of age and the primary caregiver of a child under 5 years of age in the home. Informed written consent was obtained from each participant. Figure 1

provides an overview of the sampling and study design.

Key survey topics included child latrine usage and barriers to use; main and secondary defecation and disposal sites (Table 1, steps 1–3); child cleaning practices and waste water disposal sites (Table 1, steps 4–6); ideal place of child defecation; and an inventory of price, purchase location, and satisfaction for all products, tools, and equipment owned and used by the household in managing children's defecation.

Survey responses were collected verbally, recorded on paper questionnaires by trained enumerators who were native Khmer speakers, and entered into Excel (Microsoft Corp., Redmond, WA).

### Focus group discussions.

One to 2 weeks after the survey, FGDs with six to seven surveyed caregiver participants were conducted in four study villages where survey results had shown diverse disposal practices, including positive caregiver behaviors. FGDs were designed to gain insight into consumer preferences related to child-friendly potties, diapers, and other new product designs and technologies for children's sanitation. An Internet and physical search of products currently available for children's sanitation worldwide and in markets in Phnom Penh, Cambodia, was conducted to identify a representative range of products to discuss with FGD participants (see Supplemental Table 1).

In each session, actual products were presented to participants to elicit discussion and feedback on preferred design features, perceived benefits, and potential age of use and price points. Key questions included whether participants had seen or were aware of the products presented, attractive features of each product and why, prices participants would be willing to pay for such products, and which products participants would want to purchase for what child ages and why.

Each FGD was recorded and a native Khmer speaking note taker took notes during the discussions. Informed oral consent was obtained before each discussion.

### Data analysis.

Hygienic child feces disposal was assessed based on the main site for final disposal reported by caretakers in the survey. Disposal sites were grouped into “hygienic” and “non-hygienic.” Although some researchers consider burial to be a hygienic disposal method, in this study, hygienic disposal was limited to disposal into an improved latrine where feces could be separated from human contact and the environment, in accordance with the definition used by the Joint Monitoring Program.^12^

Logistic regression analysis was used to examine associations between explanatory variables and the binary outcome of hygienic (versus unhygienic) child feces disposal, at the household (caregiver) level, as defined above (see details in Table 2). Analysis was limited to one child per household selected at random when the household had more than one child < 5 years of age (*n* = 32) (see Table 2). Explanatory variables were first tested one at a time in univariable analysis, and then those explanatory variables with a significant univariate association (*P* < 0.05) were included in a final multivariable regression model. Because child age was a major determinant of hygienic disposal, regression controlled for child age to avoid confounding due to child age differences between caregivers/households. It was not possible to control for economic status, another potential confounder, because of difficulties collecting accurate household income data in rural Cambodia. Survey data analysis was conducted using STATA13 (StataCorp LP, College Station, TX). All associations presented in results are child age-adjusted and *P* values are from the Wald test.

FGD notes were analyzed following World Health Organization guidelines.^13^ Key themes and outcomes were identified and compared with common themes and outliers for each group and across all groups.

## Results

### Household survey: participant and household characteristics.

All but one enrolled household completed the survey. Sample characteristics are provided in Table 3. Caregiver ages ranged from 20 to 74 years (median 35) and the majority (88%) was female. Child ages ranged from 1 to 60 months (median 24 months) and 53% were male. All but three participating households owned a pour-flush latrine in which water is used for anal cleansing, and 43% had owned their latrine for 2–5 years.

### Children's latrine use.

The mean age at which caretakers felt a child could use the latrine alone was 5 years, while the mean age for consistent use was 7 years.

Primary reasons expressed by caretakers for why children did not always use the latrine were that their child was too small or could not squat over the latrine pan (94%), or might fall down (12%), the caregiver was too busy (9.3%), the child was afraid (6.5%), or it was difficult to teach the child to use the latrine (5.6%). The main challenges their child was likely to experience (or had experienced) when starting to use the latrine were that they would be too small (24%), it would be hard for them to clean themselves and flush (20%), the latrine was too slippery (20%), the child would be afraid (17%), it was hard for the child to squat (16%), and it took too much time to wait with the child (16%). When asked what help caretakers needed to provide for their child to use the latrine, most said they needed to clean the child's bottom (73%) and pour flush the latrine (52%).

### Child cleaning and handwashing.

Caretakers reported cleaning their child immediately after defecation (67%) or after first disposing of the feces (33%). Water used to clean the child was thrown in the yard (43%) or washed directly into the latrine (28%) in most cases. Most caretakers reported washing their hands immediately after cleaning their child (80%); less than 1% of households said they did not wash their hands at any point. Although most caretakers reported using soap to wash their child (78%) or their hands (94%), in the spot checks, there was often no soap present around water sources. In addition, no disinfectant supplies were identified during the survey that might be used to clean child feces management equipment.

### Children's feces management practices.

Step 1. Defecation: main sites of children's defecation were the latrine (31%), a traditional potty (29%), the yard (20%), a disposable diaper (13%), or a reusable diaper (4%).
Step 2. Transportation: primary methods used to move feces when defecation occurred outside the latrine were traditional potties (38%), shovels (24%), scoops (11%), plastic bags (11%), or diapers (7%). Less than 10% reported using pans, trash, cardboard, buckets, baskets, or hands.
Step 3. Disposal: main sites of children's feces disposal when the latrine was not used for defecation (69%) were the latrine (37%), burial (20%), or the garbage (9%). Less common were the woods, a drain or ditch, and burning.

Overall, 63% of households reported defecation or disposing of feces hygienically in the latrine as their main practice for the randomly selected child in their home. However, over 50% of households reported using alternative disposal sites in addition to the main site. If hygienic feces disposal is defined as the practice of households who only ever use a hygienic disposal site, only 36% of households consistently disposed of children's feces hygienically.

**Figure 2.**
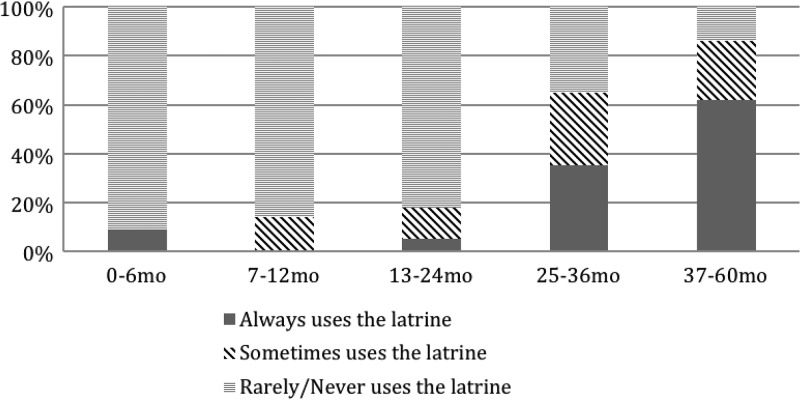
Latrine use by child age.

Place of defecation and final disposal site varied depending on child age. Up until 2 years of age, less than 10% of children were reported to “always” use the latrine, although two children less than 6 months were reported to “always” use it by being held over the latrine. At 2 years of age, the proportion of children “always” using the latrine increased substantially to 35%, and reached 62% after 3 years of age (see Figure 2

).

**Figure 3.**
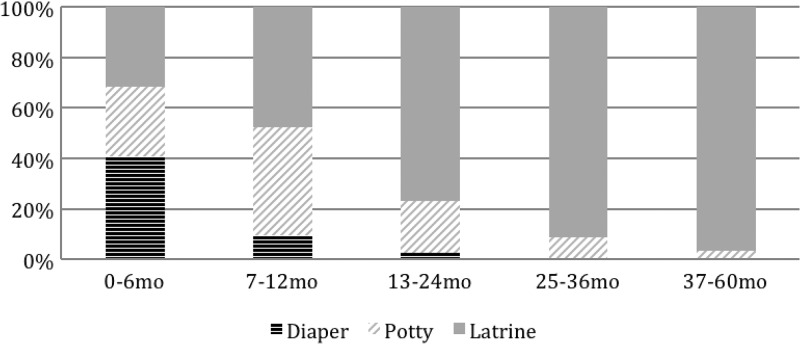
Ideal place of defecation by child age.

The ideal place of defecation for children 0–6 months of age, as reported by caretakers, was the diaper (41%); for children 7–12 months of age, the traditional potty (43%) or latrine (48%); and for children over 12 months of age, the latrine (87%) (Figure 3

). Traditional potties are made of metal or plastic and are used as chamber pots at night by most household members. The latrine was viewed as ideal primarily for being “hygienic or clean” (63%), while the traditional potty was viewed as ideal because it was “age appropriate” (44%) and the diaper because it was “easy to clean” (64%).

Children aged 7–12 and 13–24 months were most likely to defecate in the yard or elsewhere in the open (38% and 33%). Younger children 0–6 months of age were most likely to use diapers while children over 2 years of age were most likely to use latrines. Potty use was highest at 13–24 months of age (41%) but remained prevalent up to 59 months of age.

**Figure 4.**
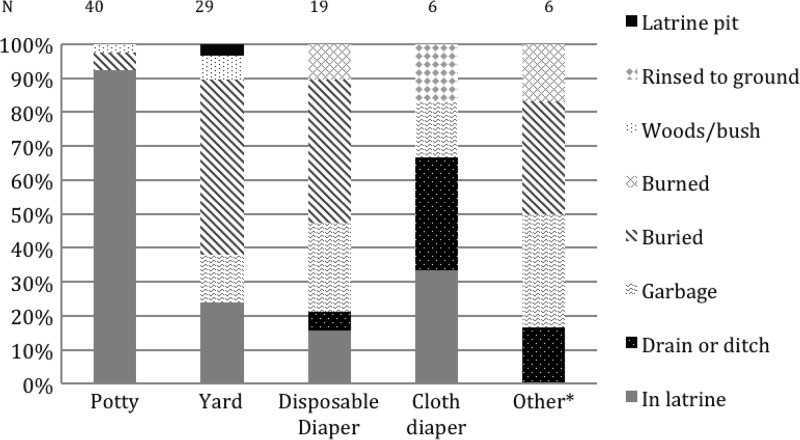
Main disposal place by place of defecation of those who reported that their child ever defecated outside the latrine (*N* = 100). *Other: on paper towels, in the child's clothes, in the forest or a field, or on furniture.

Final disposal of children's feces depended on defecation place (Figure 4

): 93% of children who defecated in potties had their feces disposed of in the latrine, compared with 20% of all those who defecated elsewhere (x^2^ *P* < 0.0001). Feces captured in cloth diapers were more often disposed in the latrine (33%) than those captured in a disposable diaper (16%), but subsamples were too small to measure a significant difference. Of those who reported transporting feces, only 29% moved feces with a shovel and 8% used a scoop to deposit them in the latrine; rather shovels and scoops were the tools most likely to be used to bury feces (65% and 67%, respectively). Many participants cited the latrine filling up or clogging with the dirt picked up by shovels and scoops as reasons why they did not use them to dispose in the latrine.

### Products currently used for children's sanitation.

Products in surveyed homes identified for defecation and feces transport included reusable and disposable items. Purchased reusable items included traditional potties (70% of households), shovels (28%), scoops (13%), brooms and pans (4%), and reusable diapers (19%). Consumables included disposable diapers (71%), wet wipes, paper towels, and cotton and plastic pads (16%). One in 10 households had a homemade item (scoop or diaper). Mean number of IYC feces management products owned per household was 2.3 (standard deviation: 0.89). Of these products, only reusable or disposable diapers appeared to be dedicated specifically for child sanitation; traditional potties were designed for and used by multiple generations in the household, particularly at night, and shovels, scoops, brooms, and pans served other purposes.

Caretakers who used commercially purchased potties, scoops, disposable diapers, and reusable cloth diapers had high rates of product satisfaction (95–100% “satisfied” or “very satisfied”). Among those who used homemade products such as cloth diapers and scoops, satisfaction was lower (73% and 0%, respectively). Of those who used shovels, 81% were satisfied.

**Figure 5.**
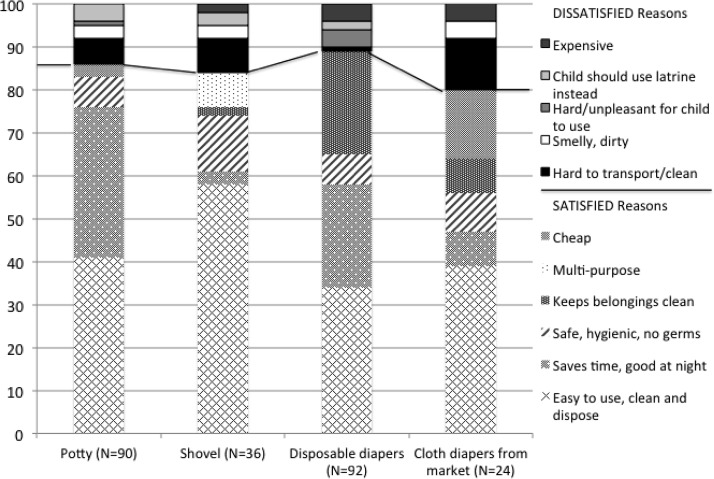
Reasons for satisfaction/dissatisfaction with current products by type.

Primary reasons reported by caregivers for their satisfaction with existing products included that they were easy to use to dispose of feces and clean; they saved time, especially at night; they were safe and hygienic; they kept the household clean; they were multipurpose; and they were cheap (Figure 5

). The main reasons reported for dissatisfaction included that tools were expensive, they were difficult to use to dispose of feces or to clean, they were smelly or dirty, or the caregiver disliked having to use any tool as the child should use the latrine.

The majority of all products (89%) were purchased at a nearby permanent market. The majority of potties, disposable diapers, brooms, and dustpans were bought less than 1 km away, while the majority of shovels, scoops, and cloth diapers were purchased 1–5 km away. Shovels were the most expensive items on average (mean US$3). Prices for reusable products varied from US$0.11 per unit (reusable diaper from market) to US$9.75 per unit (potty) while for disposable items they varied from US$0.10 (cotton pad) to US$2.20 (high-quality disposable diaper). Disposable diapers were the lowest cost child sanitation product per unit (mean US$0.43).

### Predictors of hygienic child feces disposal.

Child age was strongly associated with a hygienic main site of disposal, with increasing rates of hygienic main site disposal observed in older age groups (Table 4). Children over 3 years of age compared with infants 0–6 months of age had 45 times the odds of having their feces disposed of hygienically (odds ratio [OR]: 45.3, 95% CI: 10.5–653.1).

After adjusting for child age (AFCA), factors significantly associated with hygienic child feces disposal in univariate analyses (Table 4, columns 1–3) included the age of the caregiver, presence of items for child feces management in the latrine, how long households had owned their latrine, and consistency of adult latrine use. Latrine ownership of 6–10 years, compared with shorter and longer ownership, had the highest odds of hygienic disposal (AFCA OR: 24.0, 95% CI: 3.29–175), while young caretakers (aged 18–27 years) were less likely to practice hygienic disposal than older caretakers, with caretakers aged 28–37 years having the highest odds of hygienic disposal relative to young caretakers (AFCA OR: 6.6, 95% CI: 1.9–23.8). Households observed with a child feces management item in their latrine had nearly four times the odds of hygienic disposal compared to those without (AFCA OR: 3.9, 95% CI: 1.2–12.4), while those who reported that adults in the household only sometimes, rather than always, used the latrine had lower odds of hygienic disposal (AFCA OR: 0.09, 95% CI: 0.01–0.95). When these variables were included in a mulitivariable analysis (Table 4, columns 4–6), the relationships described above remained consistent, but the *P* values for item presence, adult latrine use, and some caregiver age groups rose to between 0.05 and 0.20.

Though not statistically significant, a number of other interesting relationships were identified during analysis (see Table 2). Access to piped water in the house or yard increased the likelihood of hygienic disposal, but this relationship was not significant before or after AFCA. Households less satisfied with their latrine were less likely to dispose of feces hygienically. Caretakers with higher levels of education, households with a higher ratio of adults to children, and households in peri-urban areas were more likely to dispose of feces hygienically, but associations were not significant.

### Focus group results.

Focus group participants were excited about the sample products (see Supplemental Table 1), and the majority had never seen products with these design features before, in particular, “modern” potties designed specifically for toilet training young children, in contrast to the familiar traditional Cambodian generic chamber pot design. Interest in products depended on child age, but the child-friendly potties were very popular in all groups and the reusable diaper covers with cloth inserts received mixed but generally positive responses (Table 5). The Safe Squat (Innovations for Poverty Action, New Haven, CT),^17^ a product designed to promote early latrine use by reducing barriers to small children using dry pit latrines in Kenya, was considered difficult to use and was not popular in any group, but this may be because the prototype presented was neither a commercial product nor designed with user input specifically for Cambodian pour-flush latrines. Attractive features of the reusable diapers were the waterproof covers and absorbent pads that allowed for infrequent and independent washing, fast drying and their price. Diaper materials were valued for duration of use, and preferences differed for frequent urination (absorbency) versus multiple defecation events (durability). For the potties, key attractive features were a wide, stable base and back rest, removable pan with cover, and easily cleanable plastic material that provide increased stability when the child is seated, even for very young children, and ease of disposal and cleaning. Participants expressed a low price point for reusable diapers (from US$0.25 to US$2.50 per diaper) compared with potties (US$2.50 to US$7.50).

Several common themes emerged in terms of consumer preferences, including versatility (a product could be used for children continuously as they grow or for both children and the elderly), convenience, time savings (a product could free the caregiver to do other things and could be used in a variety of locations), ease of cleaning, and cost savings.

Attractive design features identified in the FGDs for reusable diaper covers with cloth liners included lightweight and easily dried fabric, adjustable sizes for multiple child ages, and attractive and stain-disguising colors. For potties, they included a wide back and base to increase stability, a removable feces pan and pan cover, and a handle to facilitate transport. A complete list of design features and suggested products for development for each child age from 0–60 months can be found elsewhere.^14^

## Discussion

### Explaining hygienic disposal practices.

The rate of hygienic child feces disposal among study caregivers (63%) was very similar, though slightly lower than national findings (70% of those using an improved sanitation facility).^7^ The rate of access to piped water on the premises in this study (53.5%) was considerably higher than the national access rate during the rainy season (12%),^7^ and may facilitate hygienic disposal,^15^ though the positive association was not statistically significant in this study.

Overall, the youngest caretakers, those with the youngest children and those with the newest latrines, were the least likely to dispose of feces hygienically, indicating that experience and habit building may influence hygienic disposal. Caregiver education has been reported as a driver of hygienic behaviors such as handwashing,^16^ but the positive association with hygienic disposal was not significant in this study. This may be due to limited educational differences or the limited study population. Caregivers represent potentially important target groups for infant and young child sanitation interventions aimed at preventing child feces from coming into contact with the environment.

Households that had owned a latrine for 6–10 years were 24 times more likely to dispose of feces hygienically, controlling for child age, than households that had owned a latrine for under a year. This association somewhat weakened but was still positive for households that had owned a latrine for over 10 years. Older latrines may have been built differently, caretakers long accustomed to latrine ownership may not have formed hygienic disposal habits, or households that built a latrine longer ago compared with more recently may have unobserved characteristics associated with lower odds of hygienic disposal.

Older caretakers were more likely to practice hygienic disposal, and those aged 28–37 years were most likely to dispose hygienically. Consistency of adult latrine use was also associated with hygienic disposal for children. This finding suggests that adults who prioritize hygienic practice for themselves may be more likely to extend this practice to their children. Finally, the presence of children's feces management equipment and items in a latrine was strongly associated with hygienic disposal. Although this does not prove a causal link between ownership of enabling products and hygienic disposal, it suggests that those who practice hygienic disposal in the latrine are more likely to have an enabling product that they leave in the latrine after disposal. The majority of items present in latrines were traditional potties, highlighting their particular role in hygienic disposal.

### Latrine use.

The increasing rates of children over 2 years of age using a latrine, and the near-universal selection of the latrine as the ideal defecation place for children over 2 years of age, suggest that interventions to increase latrine access could increase young children's use of latrines.

Barriers to children using the latrine were mainly based on the child age and size and the time a caretaker is required to spend with small children when they begin using latrines. Study findings on barriers to hygienic disposal were similar to those identified by Gil and others,^3^ which included the time and energy required for disposal, inaccurate perceptions of the harmlessness of children's feces, limited resources, and perceptions of dangers surrounding latrine use for young children. Although children under 2 years of age were the least likely to use the latrine, caretakers felt that a child would not be able to use one without assistance until 5 years of age, and not consistently until 7 years of age, with some participants citing ages up to 12 years. This implies that even children over 5 years of age may experience barriers to consistent latrine use not identified in this study. The Safe Squat^17^ was not a popular product concept with Cambodian participants as presented in the FGDs. However, further research into more appropriate designs for Cambodian pour-flush latrines such as seats fitted over pans or improved child-friendly and safe potty designs that allow caretakers to do other tasks while the child is seated and are quick and easy to clean, could be implemented to overcome some of the identified barriers to small children using latrines.

### Consumer interest and product design.

The study found high levels of ownership and interest in products for IYC feces management in Cambodia among latrine-owning households. In general, products were purchased close to participants' homes and at prices participants were willing to pay, indicating that current market conditions are amenable to the supply and sale of hygiene management products in local markets. Some products, such as shovels and scoops, were valued as multipurpose tools, while traditional potties were not child friendly and designed as chamber pots for general use by household members, particularly at night. Diapers were used exclusively for managing children's feces. FGD participant interest in the new sample products revealed a latent demand among caretakers for better-suited and more child-friendly products designed specifically for IYC feces management, which were unavailable to study participants in local markets.

The appeal and uptake of new dedicated IYC feces management products will depend on their price and utility: consumables, such as disposable diapers, can only be used once, while potties may represent a higher cost but can be used for multiple years if designed for adaptation as a young child ages.

### Scoops and shovels.

Our finding that shovels and scoops were much less likely to be used to safely dispose of feces in the latrine because of the potential for pour-flush latrines widely used in Cambodia to clog with dirt indicates that safe child feces disposal interventions promoting scoops^18^ may not be appropriate for the Cambodian context. Ideally, a tool should prevent children's feces from ever coming into contact with the environment, as with a diaper or potty. A scoop or shovel may facilitate hygienic disposal, but was most commonly used to bury feces in this study, a practice that was not considered hygienic as rains and animals had a high likelihood of uncovering the feces. However, for households with no latrine, a scoop may serve as a solution to ensure caretakers are not directly contacting feces and feces are removed from the immediate household environment.

### Potties and reusable diapers.

The results show the potential for well-designed child-friendly potties and reusable diapers to facilitate hygienic disposal in the age groups least likely to have feces disposed of hygienically. The link between potties and hygienic disposal is consistent with other study findings.^19^,^20^

For potties, FGD participants cited their ease of use, transport, and cleaning, in addition to the time saved, especially at night, as key advantages. These preferences were consistent with the survey findings on caregiver satisfaction with potties (Figure 5). The FGDs identified the stable base, back, pan cover, removable feces pan, and lightweight, easily cleaned material as key design features for child-friendly potties dedicated for IYC feces management. The sample potty features offered time savings compared with current practice. Participants noted that with the stable base of sample potties, they could engage in other household tasks while their children used the potty, rather than having to hold and wait with the child. In addition, ease of disposal into the latrine represents energy savings compared with burying feces. Research in other countries has shown that children may reject potties if mothers try to force them to use them at a young age (under a year) or if they have experiences of falling off them.^20^ The ability of IYC dedicated potties to be used at multiple child ages was also important to participants, as children often use a traditional potty at night even after they are old enough to use the latrine during the day. Thus, product designs will need to address consumer preferences for use at multiple ages. Given the high rates of safe disposal and high levels of familiarity and satisfaction for general-purpose potties, investing in promotion of increased potty uptake and proper use of improved IYC potty designs for the Cambodian market has significant potential to improve hygienic practices.

For the reusable diaper covers with pads, key advantages were the cost savings, as well as the ease of use, disposal, and cleaning. However, many participants felt it was hard to dispose of feces with these diapers and clean them. In our sample, a large number of households reported using disposable diapers, in contrast to a relative absence of mention of these products in previous studies.^3^ Disposable diapers were highlighted as a time-saving device during the night in the FGDs, and it is probable that ideal feces management practices for Cambodian households with a latrine (which is typically located outdoors) will require different products for use at night compared with the day. Disposable diapers can be a hygienic method of managing children's feces if properly disposed of, through burning or sanitary trash disposal. Disposable diapers were less likely to be hygienically disposed than reusable diapers (Figure 4), and are thus not the ideal primary defecation product. Reusable items could be used during the day while consumables such as disposable diapers could be used at night.

Compared with the current practice of using disposable diapers, reusable diapers require increased time and energy to wash and dry. This barrier is partially countered by the cost savings of reusable diapers, but the price point participants named was still quite low, and the long-term savings of reusable diapers may need to be emphasized in marketing. In addition, reusable diapers are not always washed into the latrine, and the exposure risk of washing them into the yard presents a challenge for improving hygienic practice. Behavior change messaging around proper wastewater disposal and handwashing promotion could be incorporated into marketing campaigns to increase impact on hygienic practice.

Participants found that both the sample potties and reusable diapers looked “new” and “modern,” and a focus on these themes in marketing may facilitate uptake. Participants were aware of varying quality between products, and could cite the price of a high- versus low-quality diaper or potty. In addition, homemade products such as diapers and scoops had the lowest overall satisfaction rates among consumers, despite sharing similar designs to the market versions, indicating consumer preference for commercially produced products. Thus there may be potential for a high-quality product to overcome low price point barriers.

Finally, easily cleaned, affordable enabling products such as child-friendly potties and well-designed reusable diapers may overcome some of the identified barriers to early latrine use, such as time spent waiting with the child and the inability for small, young children to use latrines, facilitating safe disposal at a younger age. Even in households without an improved latrine, potties and diapers may facilitate the separation of feces from contact with the household environment and caretakers' hands.

### Hygiene practices in the feces management process.

A challenge to the promotion of hygienic feces disposal practices will be ensuring the primary caregiver uses consistent hygienic practices in each step in the disposal process (Table 1), each of which represents an additional burden of time and energy to caretakers.

Although products have the potential to facilitate hygienic feces disposal, they also may increase household contamination through improper cleaning, wastewater disposal, and lack of handwashing. Hygienic tool cleaning practices included using basins to wash diapers and brushes to wash potties into latrines. No consumable cleaning products or disinfectants, such as bleach, were observed.

It is important to note that while all but one household reported washing their hands after child feces disposal, many caretakers said they did so as a part of the child-cleaning process, which others have found does not always involve soap.^3^ Caretakers who wash their hands after cleaning the child but before disposing of the child's feces may not wash them again before moving to another task.

Several studies have demonstrated the importance of access to sufficient quantities of water for the practice of hygienic behaviors, such as handwashing.^21^ Access to water did not appear to be a barrier for households in this study, as the majority had on-site access to piped water. The rate of piped water access in the study was much higher than Cambodia's national access of 12% (wet season) and 14% (dry season).^7^ Villages with high levels of latrine coverage may also have higher levels of piped water coverage. The high level of piped water coverage in study villages presents a limitation of study generalizability. However, the practice of unhygienic children's sanitation behaviors found in this study of the most favorable piped water and sanitation access conditions indicates that practices are likely to be even worse in areas with lower access.

Households without a latrine are generally among the poorest and most vulnerable populations, and further research into facilitating hygienic feces management among those without latrines may be needed.

### Study limitations.

Practices were measured using self-report, which may yield biased results as participants are likely to report practicing “good” behaviors—such as handwashing with soap—more frequently than they actually do.^22^ However, as the study was exploratory, these errors are understood to be present and have been considered when looking at the implications of the data. The small sample size and purposeful rather than random village selection limits the generalizability of the data. In addition, as villages were included only if they had over 80% coverage of improved sanitation facilities, they differ from other villages in rural Cambodia in terms of incomes and access to markets—most villages with this level of sanitation coverage were located along or near major roads or outside larger towns. These limitations also extend to the generalizability of findings from the FGDs, as they were conducted in the same villages. Though socioeconomic and other factors were not analyzed as a part of this study, their impact should be considered when interpreting the significance of the study findings and their generalizability.

## Conclusions

The study findings show a strong need for children's sanitation interventions in Cambodia targeting primary caregivers of children under 5 years of age, and particularly children under 2 years of age, who are the least likely to use the latrine and the most likely to have their feces disposed of unhygienically. On the basis of these findings, child-friendly potties and diapers have been identified as the tools that can facilitate caregivers' hygienic management of children's feces in Cambodia, while shovels and scoops are less likely to facilitate disposal into a latrine. In this context, hygienic management implies that feces do not come into contact with the environment; are transported in a device that facilitates easy disposal in the latrine without direct contact with hands; and are disposed of in an improved latrine. Products should be easy to clean, and additional materials and equipment may be needed to ensure that the child can be washed, wastewater can be disposed of hygienically and caretakers wash their hands.

The findings of this study underline the potential impact of children's sanitation products on the hygienic management and, in particular, the hygienic disposal of children's feces in Cambodia, and similar contexts elsewhere.

## Supplementary Material

Supplemental Datas.

## Figures and Tables

**Table 1:** The six steps of child feces management and disposal and associated hygiene behaviors and products

Disease transmission behaviors	Disease prevention behaviors	Unhygienic tools/place/practice	Hygienic tools/products
Step 1:			
Defecation site
Feces enter the environment through open defecation	Safe containment of feces through use of an improved latrine or capture in age-appropriate hygienic containment product	Yard, furniture, and paper towels	Reusable or disposable diaper, potty, and latrine
Step 2:			
Feces transport
Feces come into contact with caretaker's hands when moved	No direct contact with caretaker's hands, through use of tools such as shovels, or hygienic capture	Hands, leaves	Feces capture in reusable or disposable diaper, potty
Step 3:			
Feces disposal
Feces left in the environment or disposed of unhygienically	Disposal of feces into an improved latrine or adequate burial or treatment; contaminated disposable materials adequately burned or treated	Buried, garbage thrown in drainage, left in yard	Latrine (direct or from potty, reusable or disposable diaper)
			Burned or sanitary garbage disposal (disposable diaper)
Step 4:			
Cleaning tools
Tools not cleaned or wastewater disposed of in the yard or open environment	Equipment (e.g., potties, shovels, and reusable diapers) cleaned with soap, wastewater disposed of in an improved sanitation facility, and items disinfected through direct exposure to sunlight	In yard, into open drain	In basin, emptied into latrine (ideally using soap), tools/diapers dried in direct sunlight
Step 5:			
Cleaning child
Child not cleaned or wastewater disposed of in the yard or open environment	Child cleaned with soap and wastewater disposed of in an improved sanitation facility	In yard, in basin emptied into yard, into open drain	Over latrine, in basin emptied into latrine
Step 6:			
Handwashing with soap
Caretaker's hands not washed after child bottom washing or any point of contact with child's feces	Caretaker's hands washed with soap after child bottom cleaning and any contact with child's feces before new activity	Not washed at key contact points	With soap after transport, disposal, and child cleaning

**Table 2:** Binary logistic regression analysis of explanatory variables of hygienic disposal outcome

Regression	Variable	Type	Definition, notes
Outcome	Hygienic feces disposal of selected child under 5 years	Binary	Yes/no: main defecation place (step 1) or feces disposal place (step 3) of child is improved latrine (child randomly selected if caregiver reports > 1 child under 5 years of age)
Explanatory	Child age	Categorical	Table 3
	Latrine ownership	Categorical	Table 3
	Caregiver age	Categorical	Table 3
	Presence of feces management tools in latrine	Binary	Yes/no: IYC feces management tool observed in the latrine during survey visit
	Adult latrine use	Binary	Adults always vs. sometimes used latrine
	Piped water at house	Binary	Yes/no
	Satisfaction with latrine	Binary	Caregiver satisfied vs. dissatisfied
	Caregiver education	Categorical	Table 3
	Adult/child ratio	Scalar	(No. of adults > 17)/(no. of children < 5) in the household
	Peri-urban village	Binary	Yes/no (vs. rural village)

IYC = infant and young child.

**Table 3:** Participant and household characteristics

Variable	Categories	*N*	%		
Caretakers (*N* = 129)					
Caregiver age (years)	18–35	63	49		
	36–49	23	18		
	≥ 50	43	33		
Caregiver gender	Female	114	88		
Households (*N* = 129)					
Number of years of latrine ownership	≤ 1	10	7.8		
	2–5	56	43		
	6–10	35	27		
	11–20	24	19		
	> 20	4	3.1		
Latrine type*	Pour flush with pan	102	80		
	Pour flush with pedestal	22	17		
	Dry pit	3	2.4		
Latrine pan material*	Ceramic	123	97		
	Other	4	3.2		
Distance from house to latrine* (meters)	0–3	59	47		
	4–6	34	27		
	7–10	15	12		
	> 10	19	15		
Satisfaction with latrine	Very satisfied	45	35		
	Satisfied	62	48		
	Dissatisfied	22	17		
Frequency of adult latrine use	Always	124	96		
	Sometimes	5	3.9		
Animals in/around house*	Yes	105	83		
Children under-five (*N* = 145)					
Child age (months)	0–6	22	15		
	7–12	21	15		
	13–24	39	27		
	25–36	34	24		
	37–60	29	20		
Child gender	Male	75	53		
Main person responsible for caring for the child	Child's mother	82	57		
	Child's father	3	2.1		
	Child's grandmother	43	30		
	Child's grandfather	11	7.6		
	Other	6	4.2		
Water (*N* = 129)					
		Wet season		Dry season	
		*N*	%	*N*	%
Main source of water	Piped into house or yard	69	54	79	61
	Standpipe/well/borehole	22	17	25	19
	Rainwater	24	19	–	–
	Bottled water	1	0.8	3	2.3
	Surface water	13	10	22	17
Is the water source on the property	Yes	117	91	112	87
Is the water purchased	Yes	66	51	82	64
Villages (*N* = 21)					
		*N*	%		
Village density	Rural	15	71		
	Peri-urban	6	29		

* Two missing.

**Table 4:** Child age-adjusted odds of practicing hygienic child feces disposal, 95% CI, and adjusted *P* values

Explanatory variable	Categories	Univariate analysis results*			Multivariable regression model results†		
		OR (col. 1)	95% CI (col. 2)	*P* value (col. 3)	Adjusted OR(4)	95% CI (5)	*P* value (6)
Child age (months)	0–6	–	–	–	–	–	–
	7–12	5.67	1.18–27.3	0.03	6.43	1.07–38.8	0.04
	13–24	8.31	2.07–33.4	0.003	12.92	2.51–66.4	0.002
	25–36	21.7	4.75–99.4	< 0.001	16.44	2.90–93.2	0.002
	37–60	45.3	8.14–252	< 0.001	87.94	10.5–653	< 0.001
Number of years of latrine ownership		AFCA					
	≤ 1	–	–	–	–	–	–
	2–5	9.42	1.47–60.2	0.018	8.46	1.20–59.5	0.03
	6–10	24.0	3.29–175	0.002	20.29	2.45–168	0.005
	> 10	15.9	2.09–121	0.008	12.72	1.42–114	0.02
Caregiver age (years)		AFCA					
	18–27	–	–	–	–	–	–
	28–37	6.64	1.86–23.8	0.004	6.55	1.51–28.5	0.01
	38–47	2.46	0.55–11.1	0.241	3.22	0.57–18.3	0.19
	48–57	4.23	1.11–16.2	0.035	3.46	0.68–17.7	0.14
	> 58	4.81	1.28–18.1	0.020	4.03	0.91–17.9	0.07
Presence of tools in latrine		AFCA					
	No	–	–	–	–	–	–
	Yes	3.87	1.21–12.4	0.023	3.15	0.82–12.0	0.09
Adult latrine use		AFCA					
	Always	–	–	–	–	–	–
	Sometimes	0.09	0.01–0.95	0.05	0.18	0.01–2.43	0.20

AFCA = adjusted for child age; CI = confidence interval; OR = odds ratio.

* Derived from the Wald test in univariable logistic regression controlling for child age, testing each explanatory variable in a separate regression model.

† Derived from the Wald test for the explanatory variable in a multivariable logistic regression model of significant explanatory variables from univariate analysis.

**Table 5:** Focus group discussion theme matrix

Topic	FGD no.				Total (4)
	1	2	3	4	
Reusable diapers					
Save money	X	X	X	X	4
Hard to wash	X	X	X	X	4
Velcro and snaps good to adjust size	X	X	X	–	3
Diaper rash may be a problem	X	–	X	–	2
May need to be changed more often	X	–	X	–	2
Child-friendly potties					
Stable, would not have to hold the child to use	X	X	X	X	4
Cover is useful	X	–	X	X	3
Removable pan is useful	–	X	X	–	2
Easy to clean and carry	X	X	–	X	3
Child might break the plastic	–	–	X	X	2
Safe Squat					
Difficult to use	–	X	X	–	2
Safer for small children than latrine	X	–	X	X	3
Elderly people cannot use	–	–	X	X	2
Child could get hurt	X	X	–	–	2

FGD = focus group discussions.
