# Supplementary material for: Infant and Young Child Feces Management and Enabling Products for Their Hygienic Collection, Transport, and Disposal in Cambodia
Source: Am J Trop Med Hyg. 2016 Feb 3;94(2):456–65. doi: 10.4269/ajtmh.15-0423 (PMC4751965; doi:10.4269/ajtmh.15-0423)
Supplement: Supplementary file 1 [file SD9.pdf]

Supplemental Table 1
FGD sample products

Focus group participants interact with cloth diapers with removable, washable pads and snap or Velcro closures	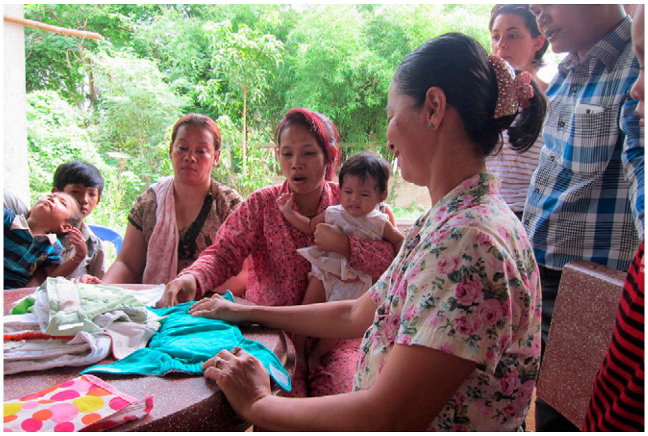
Focus group participants discuss the merits of children's potties	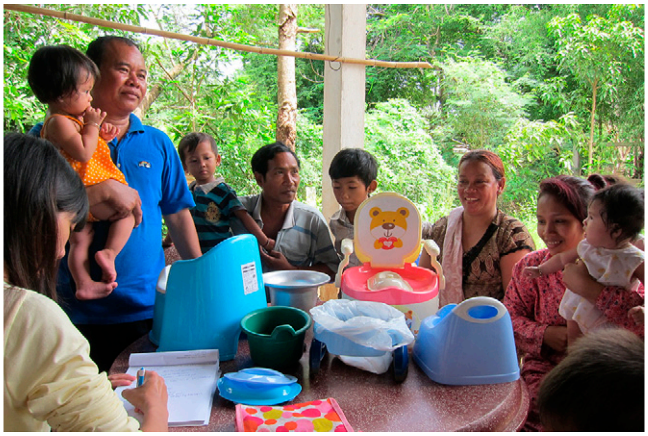
Focus group participants investigate the Safe Squat latrine cover, locally produced	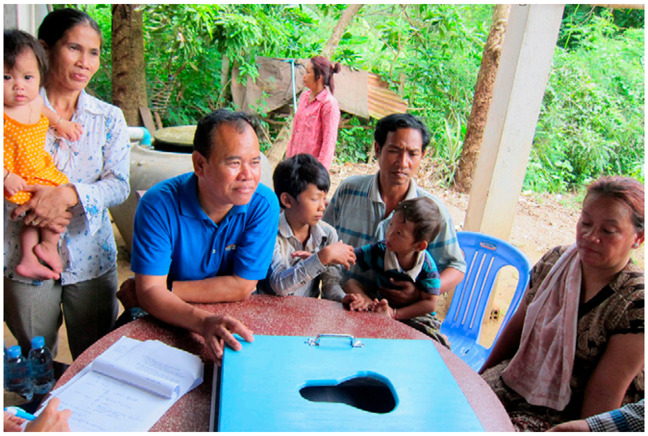

FGD = focus group discussions.
